# Transforming Aesthetic Medicine With Generative Artificial Intelligence

**DOI:** 10.1111/jocd.70015

**Published:** 2025-02-07

**Authors:** Marina Landau, Martin Kassir, Mohamad Goldust

**Affiliations:** ^1^ Arena Dermatology and Department of Plastic Surgery Shamir Medical Center Be'er Ya'akov Israel; ^2^ Worldwide Laser Institute Dallas Texas USA; ^3^ Department of Dermatology Yale University School of Medicine New Haven Connecticut USA

**Keywords:** aesthetic medicine, artificial intelligence, cosmetic dermatology, generative artificial intelligence

Dear Editor,

The combination of generative artificial intelligence (AI) into cosmetic dermatology represents a transformative advancement in patient care, bridging the gap between expectations and outcomes [1, 2]. By providing virtual simulations, generative AI is expanding consultations, procedural planning, and clinician training [3, 4].

Generative AI tools such as Modiface (acquired by L'Oréal), and Crisalix have redefined patient consultations. By analyzing high‐resolution facial scans, these platforms generate dynamic 3D models that simulate potential changes from dermal fillers, neurotoxins, or laser treatments. For instance, Crisalix allows patients to visualize the results of facial contouring or breast augmentations, helping set realistic expectations and matching with their aesthetic goals (Table 1).

**TABLE 1:** Generative AI applications in cosmetic dermatology.

App/platform	Main features
Modiface	High‐resolution facial scan analysis, dynamic 3D models for simulating dermal fillers, neurotoxins, and laser treatments
Crisalix	Visualization of facial contouring, breast augmentations, and other aesthetic procedures; helps set realistic patient expectations
AnatomyNEXT	Integration of augmented reality (AR) with AI for real‐time anatomical insights during procedures, reducing complications
Touch Surgery VR	Virtual training environments for skill enhancement; dynamic response to filler injection techniques
FAIT (Fundamentals of Aesthetic Injectable Training)	Virtual models for practicing injectable techniques; builds confidence before live patient treatments
Dermanostic	AI‐powered tools utilizing diverse datasets to enable accurate simulations for individuals with skin of color
Perfect365	Focuses on inclusivity with diverse demographic datasets for accurate aesthetic simulations
YouCam Makeup	Facilitates remote consultations with personalized assessments, expanding access to cosmetic advice
FaceApp	Consumer‐facing app for visualizing aesthetic changes; often criticized for promoting exaggerated or unrealistic beauty standards

These simulations are critical in reducing patient dissatisfaction and post‐procedure regret. By informed decision‐making, these tools also help decrease potential medicolegal conflicts. This clarity provides trust and confidence, enhancing the overall patient experience.

Generative AI applications analyze skin elasticity, volume deficits, and facial features to assist in creating customized treatment plans. For example, dermatologists can predict the cumulative effects of dermal fillers, laser treatments, and resurfacing procedures in a single simulation, optimizing procedural strategies.

Generative AI also addresses safety concerns by visualizing vascular anatomy to guide filler placement, thereby minimizing risks such as vascular occlusion. AnatomyNEXT integrates augmented reality (AR) and AI to offer real‐time anatomical insights during procedures, ensuring precision and reducing complications.

Platforms like Touch Surgery VR and the Fundamentals of Aesthetic Injectable Training (FAIT) provide virtual environments for practitioners to enhance their skills. Generative AI enhances these training programs by simulating realistic outcomes and complications. For instance, trainees can practice filler injections on virtual models that respond dynamically to their techniques, building their confidence before treating live patients.

Generative AI simulations also aid in postoperative planning by modeling recovery trajectories and potential side effects, ensuring proactive care.

Generative AI addresses gaps in inclusivity, particularly for individuals with skin of color, where traditional tools often fall short. Platforms like Dermanostic and Perfect365 utilize diverse datasets, enabling accurate simulations for patients across various demographics.

Moreover, AI‐powered virtual consultations facilitate access to care. Remote consultations, supported by tools like YouCam Makeup, allow patients in underserved areas to receive personalized assessments, expanding access to expert cosmetic advice.

While the potential of generative AI is significant, challenges remain. Algorithmic biases, data privacy concerns, and the accuracy of simulations are critical issues. For example, consumer‐facing apps like FaceApp, despite their popularity, often exaggerate results or promote unrealistic beauty standards. Practitioners need to be mindful so as to not create and set unrealistic expectations. Regulatory frameworks are essential to ensure that these tools meet clinical‐grade standards, emphasizing safety, transparency, and equity [5].

Generative AI shows promise for real‐time adjustments during procedures, enabling clinicians to refine techniques based on immediate feedback. Collaboration between dermatologists, data scientists, and regulators is vital for refining algorithms, expanding diverse datasets, and ensuring ethical implementation.

Generative AI is reshaping cosmetic dermatology by enhancing patient communication, improving procedural outcomes, and providing inclusivity. Tools like Modiface, Crisalix, and ReflectMed show the practical applications of AI in daily practice. By addressing challenges and adhering to ethical guidelines, generative AI can complement human expertise, leading to superior outcomes and transforming the future of aesthetic medicine.

## Consent

Informed consent is unnecessary for this review.

## Conflicts of Interest

The authors declare no conflicts of interest.

## Data Availability

The authors have nothing to report.
